# Symptomatic Gastrocnemius Vein Aneurysm

**DOI:** 10.7759/cureus.36518

**Published:** 2023-03-22

**Authors:** Kayla Krause, Loughlin Wylie, Tarik Ali

**Affiliations:** 1 Vascular Surgery, Penn State Heart and Vascular Institute, Penn State Health Milton S. Hershey Medical Center, Hershey, USA

**Keywords:** symptomatic aneurysm, calf pain, vascular surgery, venous insufficiency, gastrocnemius vein aneurysm

## Abstract

Gastrocnemius venous aneurysms are rare and thought to be caused by trauma or inflammation. Only three prior cases of gastrocnemius venous aneurysms have been reported in the literature. A 57-year-old female presented with severe right lower extremity calf pain. She was found to have a gastrocnemius vein aneurysm which was surgically resected. Two months after resection, the patient has no residual symptoms and was able to return to her activities of daily living.

## Introduction

Venous aneurysms are uncommon but have been described in all of the major veins [1]. They occur equally between the sexes and are seen at any age [2]. Primary venous aneurysms may be caused by inherent vessel wall weakness or have no known cause, while secondary venous aneurysms are caused by inflammation or mechanical stress [3]. However, gastrocnemius vein aneurysms are extremely rare; only three have been reported in the literature [3-5]. One followed compressive knee trauma, one presented with worsening pain when lying down, and the third presented with thrombosis. In this study, a case of a symptomatic primary gastrocnemius vein aneurysm is described.

## Case presentation

A 57-year-old female presented to our institution in December 2021 for debilitating right lower extremity pain localized to the posterior calf. Her pain was worse with walking or standing and improved by being sedentary and elevating the lower extremity. Her activities of daily living were severely restricted. She had a relevant past medical history of obstructive sleep apnea on continuous positive airway pressure, hiatal hernia, and gastroesophageal reflux disease. She has a relevant past surgical history of a cesarean section, left meniscus repair, and an abdominal hysterectomy. Her medications include aspirin, duloxetine, and omeprazole. She is allergic to Bactrim, Levaquin, succinylcholine, and Zithromax. She is a former smoker and drinks alcohol occasionally. Her family history is significant for aneurysms in her siblings.

**Figure 1:**
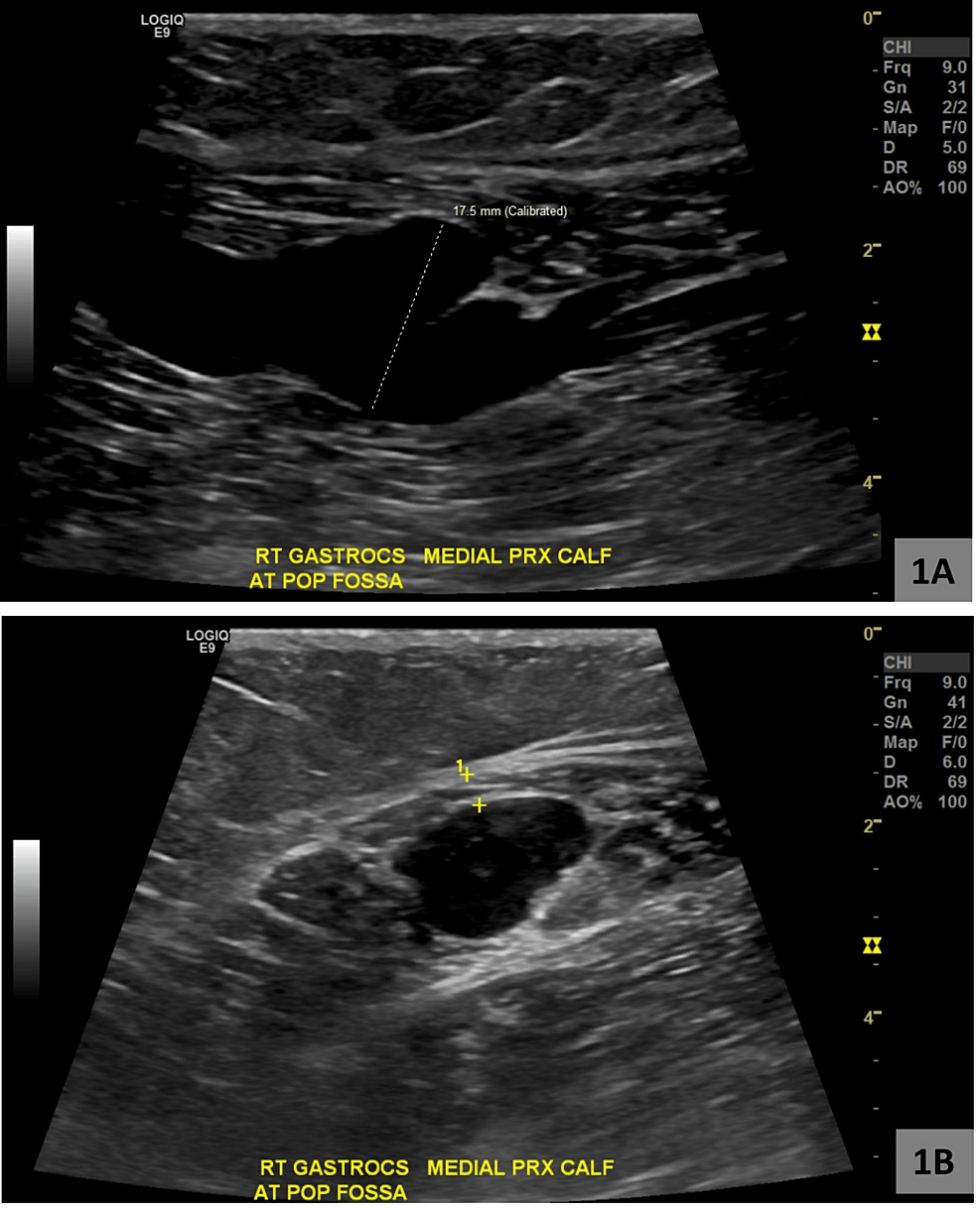
Ultrasound showing the right gastrocnemius at the medial proximal calf at the popliteal fossa A: Lateral view of the right gastrocnemius vein aneurysm, B: Frontal view of the right gastrocnemius vein aneurysm.

She previously had extensive workup by multiple specialists which revealed right lower extremity venous insufficiency with reflux in the superficial system, as well as an aneurysm of the gastrocnemius vein measuring 1.4 x 1.7 cm (Figures 1A-1B).

She tried compression with minimal relief. On presentation, she was found to have a palpable right dorsalis pedis pulse and intact motor and sensory responses in the right lower extremity. She also had significant tenderness to palpation of the right posterior calf and significant discomfort in the posterior calf with dorsiflexion of the right foot. Blood pressure was 142/93 mmHg.

On January 24, 2022, imaging showed one of two gastrocnemius veins at the proximal calf measuring approximately 1.7 cm in the sagittal plane, located approximately 1.7 cm from the skin surface.

**Figure 2:**
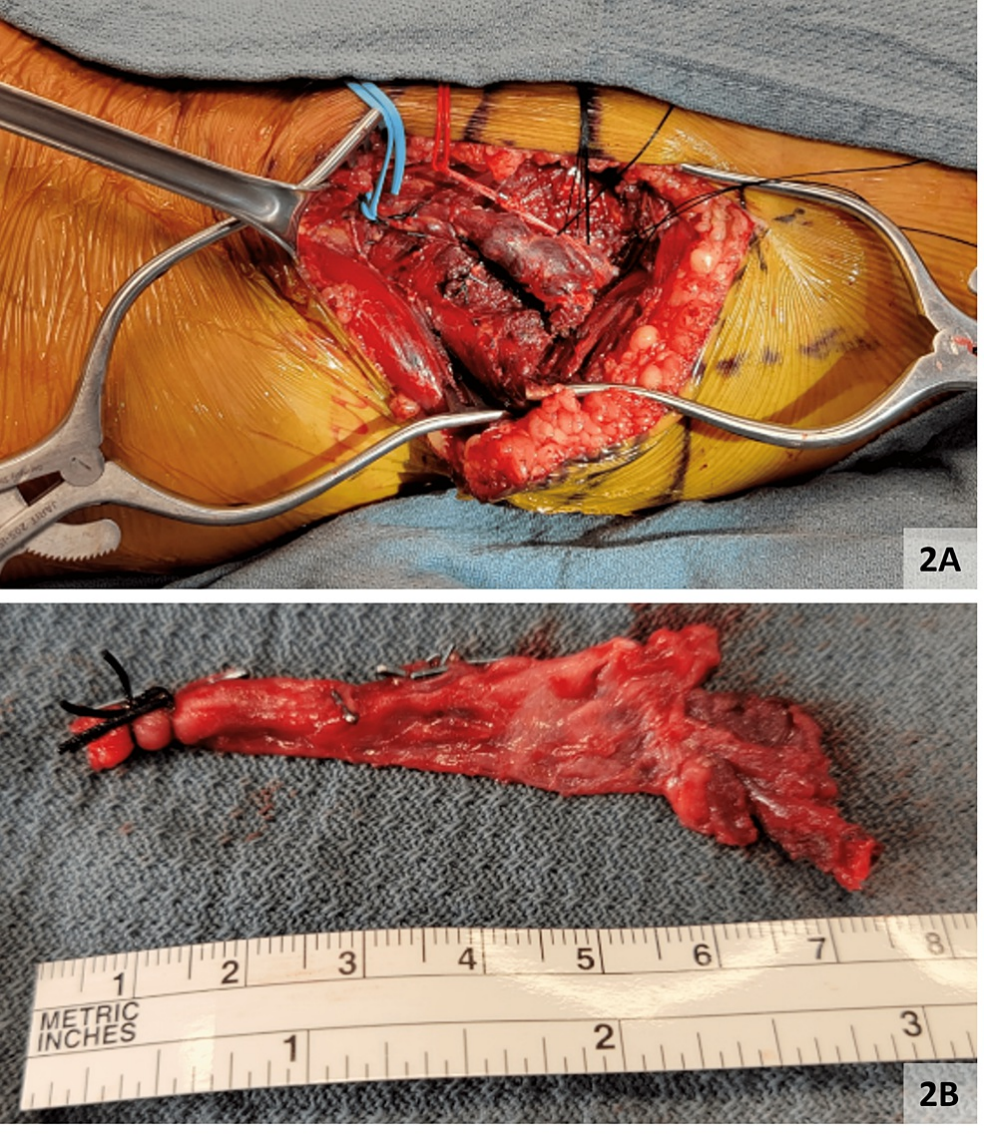
Gross images of the right gastrocnemius vein aneurysm A: Right gastrocnemius vein aneurysm visualized in the right calf, B: Right gastrocnemius vein aneurysm measuring 7 cm in length.

The right lower extremity gastrocnemius vein aneurysm was evaluated via ultrasound and its location was marked on the skin. Once the gastrocnemius vein was identified, it was circumferentially dissected. Then, distal dissection through the muscle fibers of the medial head of the gastrocnemius muscle was performed to expose the gastrocnemius vein aneurysm. The aneurysm was carefully dissected, and all side branches were tied or clipped (Figure 2A). The vein was then traced up to its confluence into the popliteal vein, where it was ligated. The aneurysm was transected and removed, and antibiotic irrigation was performed (Figure 2B).

**Figure 3:**
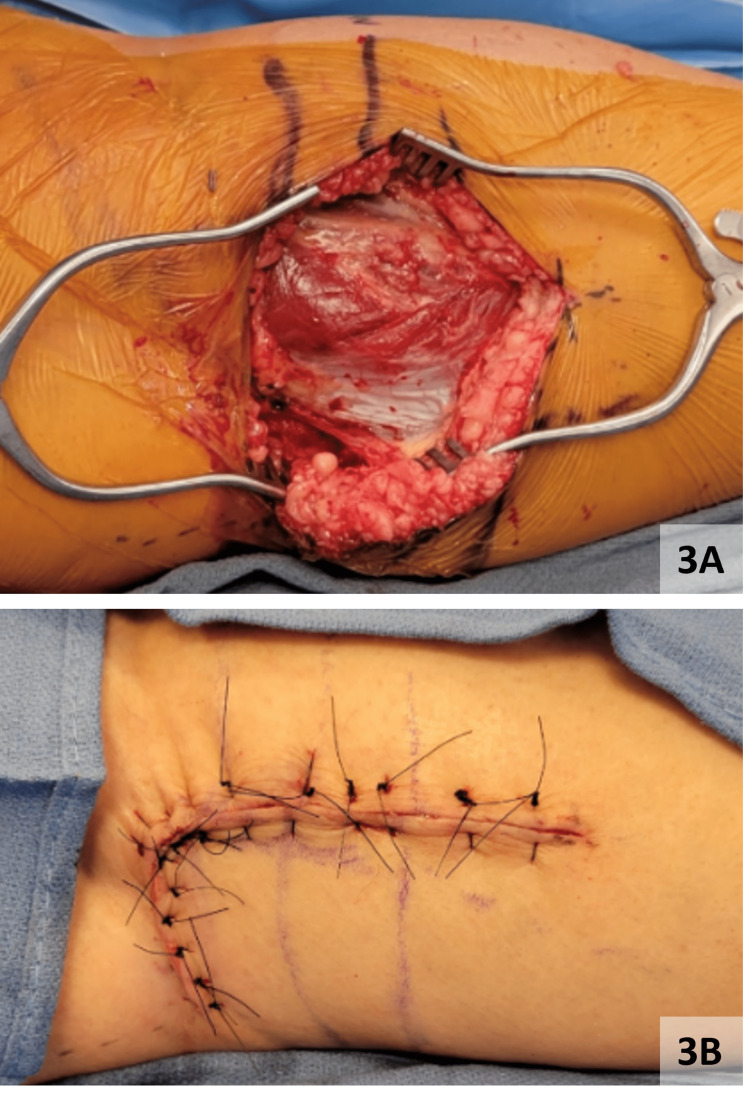
Surgical closure following right gastrocnemius vein aneurysm removal A: Gastrocnemius following medial head approximation, B: Skin closure utilizing vertical mattress sutures.

Hemostasis was achieved via electrocautery and thrombin spray in the muscular bed. The edges of the medial head of the gastrocnemius muscle were approximated, and the deep dermis and skin edges were approximated in a vertical mattress suture style (Figures 3A-3B).

At her two-week post-operative visit, the patient had significant improvement in her lower extremity pain. After two months, she reported resolution of her calf pain with a steady increase in activity level. Postoperative peripheral venous testing showed no abnormalities and subsequent lower extremity ultrasound revealed only surgical scar tissue.

## Discussion

Gastrocnemius vein aneurysms are extremely rare but as they have been found incidentally on imaging, their true prevalence may be underestimated [3]. When comparing the presented case with similar cases, all were found on imaging, and all were surgically excised with good long-term results [3-5]. Compression was not helpful in relieving symptoms. However, there are no standardized guidelines for workup and treatment in this patient population.

Despite the positive outcomes following treatment, patients frequently have weeks to months of pain prior to diagnosis which may be debilitating, interrupt sleep, and interfere with activities of daily living [4-5]. Including the current case, patients have been initially diagnosed with venous insufficiency and reflux, and ultimately saw multiple practitioners before they were able to be treated, causing increased patient morbidity and increased healthcare cost [5]. Thus, we recommend imaging and specifically bedside venous duplex ultrasound in the setting of venous reflux as a rapid and cost-effective means of ruling out this diagnosis.

While only very few may become symptomatic, symptoms are not predictable across cases. One report found that pain worsened with lying down, owing to vein relaxation and bulging in the supine state, while muscle compression with standing improved symptoms and compressed the aneurysm [5]. However, our report found that the patient was symptomatic with standing, but the pain was relieved with rest. This may be due to the size or location of the aneurysm, but either presentation may raise suspicion for this diagnosis.

Using careful dissection and tracing the aneurysm up to its inflow and outflow points prior to ligation and removal is a safe and effective means of treating a gastrocnemius vein aneurysm. However, further study is warranted on a larger sample to guide clinical best practices. This study adds to the current, limited cases of gastrocnemius vein aneurysms. With increased awareness and screening, a better estimate of the existing prevalence may be elucidated.

## Conclusions

This case adds to the scant literature on the extremely rare gastrocnemius vein aneurysm. Practitioners must have a high index of suspicion in patients with focal calf pain in the absence of risk factors and perform routine venous ultrasound in the workup and treatment of this rare pathology.
